# Multi-omics landscape of circadian rhythm pathway alterations in Glioma

**DOI:** 10.1080/21655979.2021.1947075

**Published:** 2021-07-05

**Authors:** Chang Zhang, Jiahui Xu, Lijun Chen, Xiaojie Lin

**Affiliations:** a Department of Internal Medicine, Guang Dong Second Hospital of Traditional Chinese Medicine, Guangzhou, China; b Department of Internal Medicine, Guangdong Key Laboratory of Traditional Chinese Medicine Research and Development, Guangzhou, Guangdong, China; c Department of Pediatrics, The Fifth Clinical College of Guangzhou University of Chinese Medicine, Guangzhou, China

**Keywords:** Circadian rhythm, prognostic biomarker, multi-omics, bioinformatic analysis

## Abstract

Circadian rhythm pathway was demonstrated pathological functions in glioma on single-gene level. We aim to depict the multi-omics landscape of circadian rhythm pathway alteration in glioma using bioinformatic analyses. Multi-omics data were obtained from “cBioPortal” database. Comparisons were done regarding clinical parameters, differential-expressed genes and functional annotations. A pathway index was generated using the expression data from TCGA and GTEx to quantify the general alteration level of the pathway with clinical association of circadian rhythm pathway index explored. A total of 30 genes were mapped on the circadian rhythm pathway. Genomic profile ofcircadian rhythm pathway genes exhibited distinct characteristics on multiple levels between lower grade glioma (LGG) and glioblastoma multiforme (GBM) patients. LGG patients presented significantly higher frequencies of multi-omics mutations, as well as significant clinical relevance, on single-gene level. Differential-expressed genes between LGG and GBM patients revealed different functions between subtypes that related to the alteration of circadian rhythm pathway. LGG have significantly higher pathway index than normal brain tissue, while GBM significantly lower than normal tissue (P < 0.01), indicating distinctly altered circadian pathway in LGG. Circadian rhythm pathway index correlated with the prognosis of LGG, but not GBM, patients, with higher score indicating better survival outcome (LGG: HR = 0.39, 95% CI: 0.26 − 0.59, P < 0.001). In conclusion, LGG have more multi-omics alterations of circadian rhythm pathway than GBM. Quantification of circadian rhythm pathway using pathway index demonstrated hyperactivated pathway status in LGG and correlated with the prognosis of LGG patients.

## Graphical abstract

**Figure:**
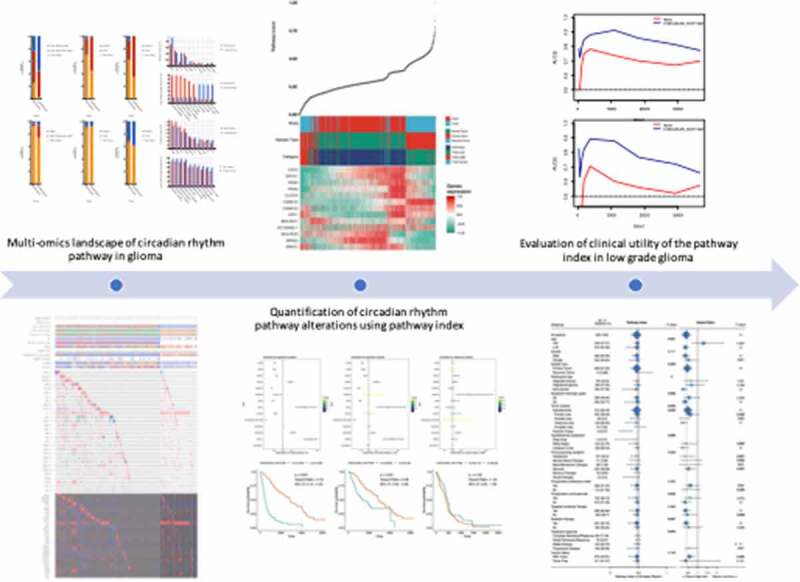

## Introduction

Circadian rhythm are high-level brain functions involving physical, mental, and behavioral changes that follow a daily cycle. Anatomical basis of circadian rhythm is found in a group of neurons called the suprachiasmatic nucleus (SCN), located in the hypothalamus above the optic chiasm, that coordinate and synchronize all peripheral clocks in the human body by translating cues from the environment into directives for the body, stimulating subsequent changes in clock gene expression so as to regulate the production of hormones that affect human behavior [1]. The cellular and molecular machinery of circadian rhythm entrainment is made of transcriptional translational feedback loops (TTFL) regulated by clock genes, including BMAL1, CLOCK, the cryptochromes (CRY 1, 2), and the period (PER 1–3). The positive and negative regulations between those factors constitute the circadian pathway.

Alteration of clock genes has long reported biological influence on aging, metabolism, immunity, DNA repair, and cell-cycle processes [2–7]. Therefore, aberrant circadian rhythms demonstrated pathogenetic correlations with various diseases, including cancer [8]. Current perspectives focusing on the mechanism between circadian rhythm disorder and carcinogenesis can be summarized as follows: (1) the expressional regulation between clock factors and oncogenes; (2) physical interactions between clock protein and oncogenic protein; (3) epigenetic modification regulated by clock genes; and (4) hormonal and metabolic alterations caused by circadian rhythm disruption [9]. Epidemiological evidence has seen correlations between shift work and prostate and breast cancer; moreover, longer duration of night shift work significantly correlates with higher grade tumor [10,11].

Glioma is the most common primary brain tumor, accounts for ~70% of primary brain tumor cases. With an annual incidence of ~5/100,000 and >14,000 new cases each year, glioma has one of the highest mortality rates among all types of cancer with the pathogenesis underlying remaining unclear. Symptoms of glioma differs by tumor location, main symptoms include visual loss, insomnia, pain, nausea, vomiting, weakness in the extremities, headaches, seizures [12]. Previous studies found intricate correlations between glioma and circadian rhythm disorders. Bioinformatic analyses demonstrated that deregulated circadian genes significantly linked to gliomagenesis, with high expression levels of circadian clock genes strongly correlated with high-grade glioma [13]. Furthermore, in vitro experiments demonstrated functions of circadian genes involving various aspects of glioma, including tumorigenesis, EMT, angiogenesis, invasion, stemness, et al [9]. CLOCK, one of the most important regulators in the circadian rhythm pathway, was emphasized significant overexpression in glioma that consequently promoted tumor cell proliferation and migration [14]. On the contrary, another core clock gene, BMAL1, was also found upregulated in brain tumor, however, inversely correlated with glioma aggressiveness by blocking of PI3K/AKT/ MMP2 pathway [15]. In addition to circadian genes, melatonin, a hormone secreted by the pineal gland which coordinates circadian rhythmicity, has also long reported tumor suppressive functions in different kinds of tumors [16–19]. In glioma, melatonin has shown anti-inflammation functions, which further lead to the immune stimulating functions in glioma [20,21]. Further exploration with melatonin as therapeutic interventions in glioma in combination with principal cytotoxic drugs found that melatonin significantly attenuated chemotherapeutic drug resistance [20].

Aforementioned studies revealed significant roles of circadian rhythm pathway in various aspects of glioma on both clinical and molecular levels. However, multi-omics alterations of circadian rhythm pathway were rarely evaluated and the landscape of circadian rhythm pathway in glioma remained unraveled. Current fast developments in bioinformatic analysis provide novel perspectives in the exploration and understanding of biology processes. By utilizing computational pipelines, identification of hub genes from RNA-Seq or Microarray data helped the experimental biologists to further carry forward the demonstration of biological mechanisms that may further benefits clinical treatments [22,23]. Furthermore, function-focused enrichment of correlated genes helped to identify putative biofactors within certain biological processes [24].

A comprehensive understanding of the underlying genetic influence and overall status of circadian rhythm pathway will enable us to understand the biological importance of circadian rhythm in glioma. Therefore, in this study, we use the multi-omics data from online databases to depict the landscape of circadian rhythm pathway and generate a pathway index quantifying the deregulating status of tumor patients by adopting bioinformatic algorithmics to fully explore the clinical significance of circadian rhythm pathway in glioma patients.

## Methods

### Data accession

RNAseq data and relative clinical phenotype information from TCGA brain lower grade glioma and glioblastoma multiforme (LGG/GBM) dataset and GTEx dataset were obtained from UCSC Xena data hub (https://xenabrowser.net/hub/) [25–27]. The expression value was shown as gene-level transcription estimates in log2(x + 1) transformed RSEM normalized count. Mutations, copy number variations, protein expression data were obtained, analyzed, and visualized using cBioPortal (https://www.cbioportal.org) [28]. Glioblastoma Multiforme and Brain Lower Grade Glioma (TCGA, PanCancer Atlas) datasets were used to construct multi-omics landscape of circadian rhythm pathway genes. For copy-number analysis algorithms like GISTIC or RAE, and levels were summarized as follows: −2 or Deep Deletion indicates a deep loss, possibly a homozygous deletion; −1 or Shallow Deletion indicates a shallow loss, possibley a heterozygous deletion; 0 is diploid; 1 or Gain indicates a low-level gain (a few additional copies, often broad); 2 or Amplification indicate a high-level amplification (more copies, often focal).

### Expressional and survival analysis

Expressional analyses were done between groups regarding both pathway index and gene expression. For measurement data, variables with normal distribution are expressed as mean (SD) and compared using the student’s t-test, variables with skewness distribution are expressed as median [IQR] and compared using Wilcoxon rank-sum test, and multi-groups comparisons were made with one-way ANOVA. For counting data, variables are expressed as n (%) and compared using the Chi-square test. All analyses were done using R package ‘ggplot2’[29]. Correlation estimates between genes and pathway index were generated using R package ‘parallel’ and illustrated using ‘circlize’[30].

Survival analyses were done using R package ‘survival’ with best separations calculated using ‘survminer’ and further adopted as cutoff. The minimal proportion of each group was set as 0.3 [31].

### Differential expression analyses and functional enrichment

Differential expression analyses were done between groups to identify genes that significantly deregulated between clusters, intersections between groups were illustrated using venn plots. Significantly differential-expressed genes (DEGs) were identified using robust rank aggregation (RRA) with integrative evaluations between multiple groups. Further functional enrichments and protein-protein interactions analysis between groups were done using ‘Metascape’ (https://metascape.org/gp/index.html) [32].

### Pathway index generation

Circadian rhythm pathway-related genes were extracted from the Kyoto Encyclopedia of Genes and Genomes (KEGG). Pathway index were generated using R package ‘Pathifier’[33], an algorithm that infers pathway deregulation scores for each tumor sample on the basis of expression data with combined normal samples from TCGA LGG/GBM dataset and normal brain tissues from GTEx dataset as the control group. Tumor samples were run with 1000 attempts for better stability with the minimal expression threshold of 4 and minimal allowed standard deviation of 0.

### LASSO regression and timeROC

LASSO regressions were done within circadian rhythm pathway related genes regarding the overall survival of TCGA LGGGBM patients using R package ‘glmnet’[34]. Cvfit plots were used for lambda value selection. The minimal lambda values were used for the construction of gene index.

TimeROC plots were used to compare the prognostic value of different signatures regarding survival data [35,36]. Analyses were done using R package ‘ timeROC’ with marginal method used for weighing calculation. Time points selected were 0, 30, 60, 120, 180, 365, 1095, 1825, 2920, 3650 days.

All statistical analyses were performed using GraphPad Prism version 8.00 for Mac (GraphPad Software, La Jolla California, USA) and RStudio version 1.2.5033 (R Core Team, Vienna, Austria) statistical software. A two-sided P-value < 0.05 was considered significant.

## Results

### Multi-omics landscape of circadian rhythm pathway in glioma

**Figure 1.**
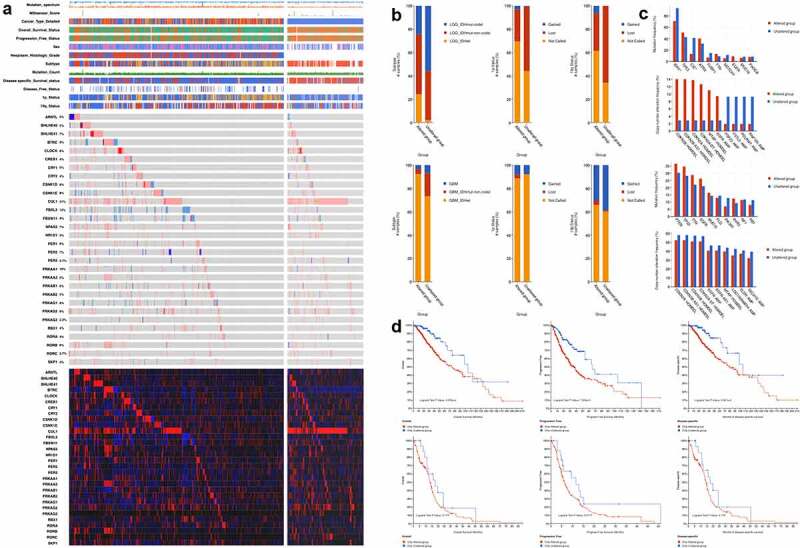
Multi-omics landscape of circadian rhythm pathway in glioma. (a). Multi-omics alteration of circadian rhythm pathway in LGG and GBM. Row: from top to bottom: clinical annotation, mutation and expression profile. Column: left: LGG subgroup, right: GBM subgroup; (b). Comparisons of sample subtypes, somatic alteration of 1p and 19q between patients with altered circadian rhythm pathway in LGG (upper) and GBM (lower); (c). Mutation and copy number alteration frequencies between patients with altered circadian rhythm pathway in LGG (upper) and GBM (lower); (d). Survival analysis between patients with altered circadian rhythm pathway in LGG (upper) and GBM (lower)

According to KEGG database, a total of 30 genes were mapped on the circadian rhythm pathway. A oncoplot was used to illustrate the mutation profiles and copy number alterations of circadian rhythm-related genes (Figure 1(a), S1). Among the 30 genes identified, CUL1 has highest mutation frequency in both LGG and GBM (21%). Comparatively, FBXL3 exhibited distinct mutational patterns between LGG and GBM patients, with most of the cases were depleted in LGG. Furthermore, LGG patients showed significantly higher frequency of co-occurrence mutation between genes, which was not seen in GBM. Among the 30 genes enrolled, CUL1, BHLHE40 and BTRC have the highest co-occurrence rate with other genes while CUL1 and RORB are the most often co-occurrence mutation (Table S1). Comparisons between copy number variation and expression data showed significant positive correlation. However, no correlation was seen between mutation and expression of the 30 genes.

Clinical characteristics between patients with alterations in the circadian rhythm pathway showed significant differences, among which, comparisons regarding sample subtypes, alterations in the 1p and 19q regions exhibited distinct alterations between LGG and GBM patients (Figure 1(b)). LGG patients have significant higher proportion of IDH non-deletion mutation in the altered group, while LGG patients have higher level of co-deletion in the altered group compared to un-altered patient, which were reversed in GBM patients. Further comparisons were done regarding the frequencies of mutation and copy number alteration between altered and un-altered patients. As shown in Figure 1(c), the mutations of IDH1 and CIC were found significantly less in the altered LGG patients (P < 0.001). Moreover, survival analyses grouped by the alteration of circadian rhythm pathway showed significant clinical relevance in LGG patients regarding both overall survive, progress-free survive, and disease-specific survive (Figure 1(d)). The alteration in the circadian rhythm pathway correlated with significant worse clinical outcomes. Comparatively, the genomic alteration of circadian rhythm pathway was significantly higher in LGG patients with higher frequency of alteration, as well as greater clinical significance.

### Functions correlated with the alteration of circadian rhythm pathway in glioma

**Figure 2.**
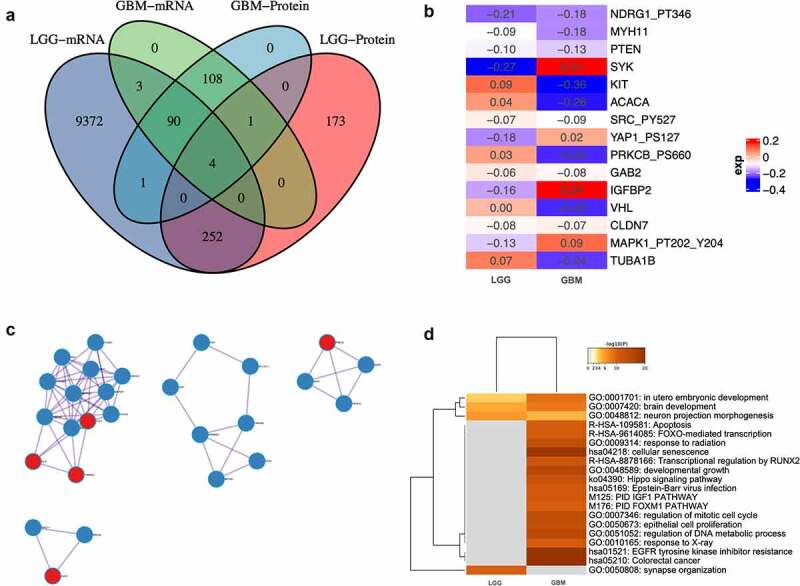
Differential analyses between patients with altered and unaltered circadian rhythm pathway as well as LGG and GBM patients. (a). Venn plot of differential-expressed genes between patients with altered and unaltered circadian rhythm pathway. (b). Differential- expressed genes between LGG and GBM patients identified using robust rank aggregation (RRA) with integrative evaluation of mRNA and protein expression; (c). Protein-protein interactions between DEGs in LGG and GBM patients; (d). Gene oncology enrichments between LGG and GBM patients

Differential-expressed genes between patients with altered and un-altered circadian rhythm pathway on both mRNA and protein level were analyzed and intersected (Figure 2(a), Supplementary Material 2). Only four genes differentially expressed in both LGG and GBM patients (ERBB1, RB1, ESR1, and KDR), indicating putative role in the regulatory of circadian rhythm pathway alteration in glioma. Furthermore, for genes that are differentially expressed in either LGG or GBM patients, integrative analysis was done with combined consideration on both mRNA and protein level. Only 15 genes altered significantly (Figure 2(b)). Interactions between altered proteins in both groups were illustrated in Figure 2(c). Intriguingly, proteins that significantly altered in GBM patients exhibited condensed cluster, while PRKCZ, CUL1, TUBB4A, DLD, and HUS1interacting with GBM proteins. Comparative functional enrichments between LGG and GBM patients revealed distinct functions that correlated with the alteration of circadian rhythm pathway. GO:0001701: in utero embryonic development, GO:0007420: brain development, GO:0048812: neuron projection morphogenesis and GO:0050808: synapse organization are enriched in LGG patients (Figure 2(d)).

### Expression profile of circadian rhythm pathway

Expression profile of the 30 circadian rhythm-related genes was extracted from TCGA LGG/GBM (Tumor = 689, Normal = 4) and GTEx (N = 1137) dataset and used to calculate the pathway index of each patients, respectively. Owing to the small sample size of TCGA normal patients, normal brain tissues from GTEx were combined to use as the normal control group.

**Figure 3.**
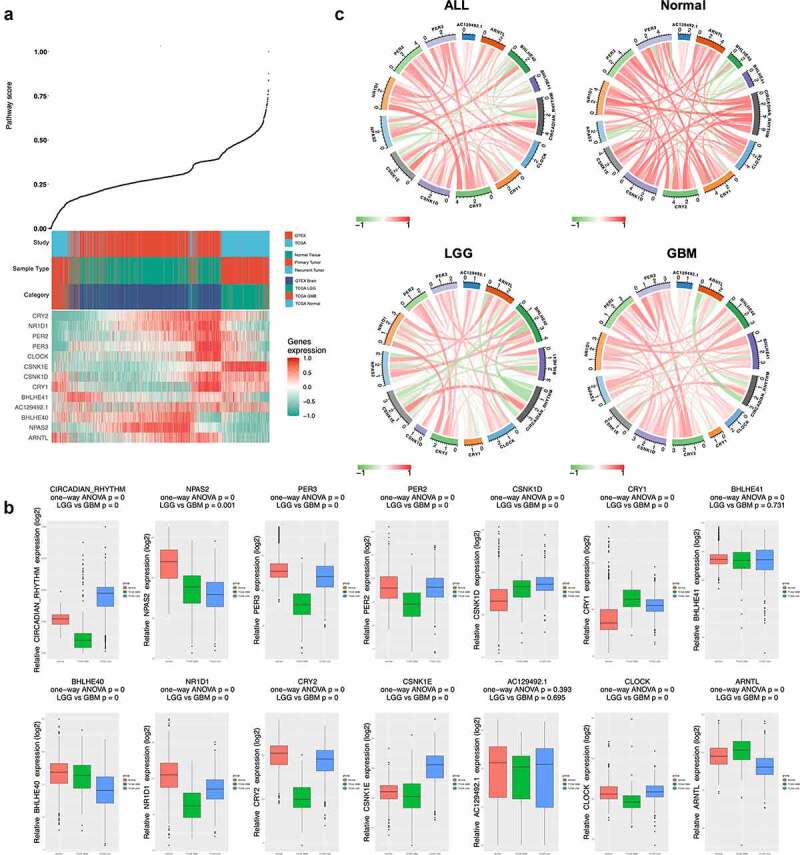
Expression profile of circadian rhythm pathway index and pathway genes. (a). Heatmap showing the expression profile of circadian rhythm pathway index and pathway genes. Patients were ranked by circadian rhythm pathway index from 0 to 1. Genes expressions were zero-centered. (b). Expression profile of circadian rhythm pathway index and pathway genes was shown with patients divided by sample group. (c). Correlations between circadian rhythm pathway index and pathway genes. Correlation R value was shown as the inner line, with green as negative correlation and red as positive

The circadian rhythm pathway index generated using only 13 genes selected, the index ranged from 0 to 1, with patients ranked accordingly, the expression profile was shown in Figure 3(a). Intriguingly, a distinct level of pathway index was seen between different pathological subtypes of glioma. Comparing to normal samples, lower grade glioma (LGG) patients has significantly higher pathway index while glioblastoma multiforme (GBM) was significantly lower, indicating that the circadian rhythm pathway was hyperactivated in LGG patients but inactivated in GBM. Comparisons focused on the circadian rhythm pathway-related genes also revealed distinct expression patterns between different sample types. Therefore, further analyses were done with tumor samples subgrouped by pathological types. As shown in Figure 3(b), aberrant expression was seen between LGG and GBM, regarding either pathway-related genes or pathway index. Among the 13 pathway-related genes, only CSNK1E showed identical expression pattern with circadian pathway index, indicating a positive correlation to the circadian pathway alteration in glioma.

To better illustrate the correlations between pathway genes and pathway index with different sample types, circos plots were used with positive correlation shown as red inner-circle line and green as negative correlation (Figure 3(c), Table S2). In normal samples, strong positive correlations were seen between pathway index and pathway genes, except for BHLHE40 and BHLHE41, which showed negative correlation in all subgroups. In contrast, CRY2 and CSNK1E showed positive correlations with pathway index in all subgroups. Differential correlations were seen in ARNTL between tumor and normal samples, with a strong positive correlation in normal group (R = 0.63, P < 0.001) while negative correlations in both LGG (R = −0.46, P < 0.001) and GBM (R = −0.60, P < 0.001) group.

### Survival analyses of circadian rhythm pathway

**Figure 4.**
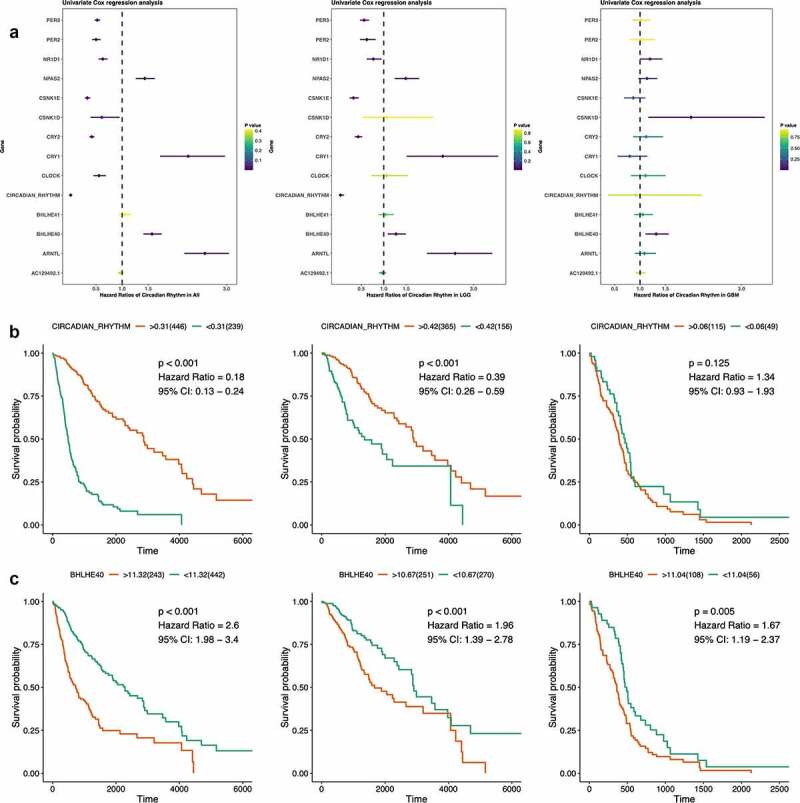
Survival analyses of circadian rhythm pathway index and pathway genes. (a). Forest plots showing the survival analyses results of circadian rhythm pathway index and pathway genes in all glioma patients and LGG and GBM subgroup, respectively. Hazard Ratio and 95% CI was show as the line colored by P value. (b). KM plot of circadian rhythm pathway index in all glioma patients and LGG and GBM subgroup, respectively. Red: high expression group; Green: low expression group. P value was log-ranked. (c). KM plot of BHLHE40 in all glioma patients and LGG and GBM subgroup, respectively. Red: high expression group; Green: low expression group. P value was log-ranked

Survival analyses were done focusing on both circadian rhythm pathway genes and pathway index regarding overall survival of glioma patients (Figure 4(a)). Significant correlations with prognosis were seen in the majority of both pathway genes and pathway index. Among 13 pathway genes, high expression of PER2, PER3, NR1D1, CSNK1E, CSNK1D, CRY2, and CLOCK was significantly correlated with better survival, while NPAS2, CRY1, BHLHE40, and ARNTL indicating worse survival outcomes. Subgroup analyses revealed similar results in LGG patients. However, most pathway genes lost prognostic value in the GBM population, which may indicate the deregulated pathway activity in different pathological subgroups.

Validations can be seen in the survival results of pathway index as shown in Figure 4(b). The circadian rhythm pathway index strongly correlated with prognosis of glioma patients, especially in LGG but not GBM patients, with higher score indicating better survival outcome (All: HR = 0.18, 95% CI: 0.13 − 0.24, P < 0.001; LGG: HR = 0.39, 95% CI: 0.26 − 0.59, P < 0.001; GBM: HR = 1.34, 95% CI: 0.93 − 1.93, P = 0.125).

Cross-sample group comparisons outlined BHLHE40 the only gene that significantly correlated with survival outcomes in all subgroups (Figure 4(c)). Higher expression of BHLHE40 indicating worse prognosis in glioma, regardless of pathological subtype, which emphasized the potency of BHLHE40 as prognostic biomarkers in glioma and a putative biological function in brain tumor.

### Clinical significance of circadian rhythm pathway index in LGG

Given the expression as well as the survival analysis results shown above, the circadian rhythm pathway index presented outstanding clinical significance in LGG instead of GBM patients. Therefore, to further explore the clinical correlations of circadian rhythm pathway index in LGG, comprehensive analyses were done regarding detailed clinical characteristics.

**Figure 5.**
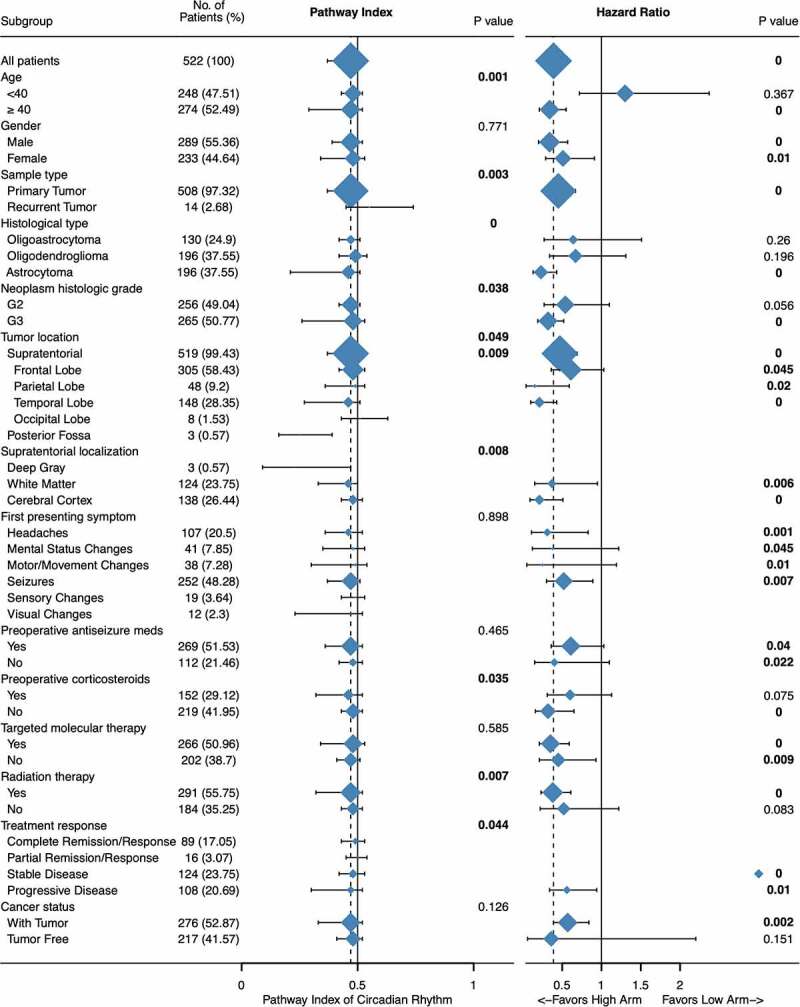
Clinical significance of circadian rhythm pathway index in LGG. Expression profile and subgroup survival analyses were done with patients divided by clinical characteristics. The expression profile was show as dot and line, representing the Median and interquartile range, while the survival analysis representing the Hazard Ratio and 95% CI. The sample size was represented as dot size and all P value < 0.05 was emphasized and considered significant

Significantly differential expression has seen in patients regarding different age, sample type, histological type, neoplasm histologic grade, tumor location, supratentorial localization, preoperative corticosteroids, radiation therapy, and treatment response. As shown in Figure 5, younger patients (< 40), with recurrent tumor, G3 or oligodendroglioma, tumor located in cerebral cortex or patients without preoperative corticosteroids or radiation therapy has higher score, which indicating distinct clinical significance of circadian rhythm pathway within tumor patients.

Combined with multi-variance subgroup survival analyses, further explorations were done focusing on unraveling the exquisite correlation between circadian rhythm pathway and clinical characteristics.

Significant differential expression was seen between old and young patients; however, clinical significance was seen only in patients older than 40 (HR: 0.34, 95% CI: 0.21 − 0.55, P < 0.001). Distinct clinical relevance was also seen between histological types, histologic grade, preoperative corticosteroids, and radiation therapy. Astrocytoma had seen the lowest pathway index comparing to oligoastrocytoma or oligodendroglioma, as well as clinical significance (HR: 0.23, 95% CI: 0.13 − 0.43, P < 0.001). Further significant subgroups including patients with G3 tumors (HR: 0.32, 95% CI: 0.19 − 0.52, P < 0.001), patients without preoperative corticosteroids (HR: 0.32, 95% CI: 0.16 − 0.65, P < 0.001), and patients undergoing radiation therapy (HR: 0.38, 95% CI: 0.23 − 0.61, P < 0.001).

Given the biological molecular machinery of the circadian clock, which is controlled by the suprachiasmatic nuclei that based in the hypothalamus, above the optic chiasm. Tumor location was considered as an important factor. However, both expression profile and subgroup survival analyses revealed no difference between different location. Tukey’s multiple comparisons test showed only marginal significant differential expression between frontal lobe and temporal lobe (0.449 ± 0.142 vs 0.406 ± 0.160, P = 0.0179), while in all groups, circadian rhythm pathway index all presented clinical significance, with higher score correlating with better overall survival.

### Prognostic value of circadian rhythm pathway index

To consolidate the prognostic value of circadian rhythm pathway index in glioma, traditional LASSO regression was done with 13 circadian rhythm pathway genes to construct a new gene index and further comparisons were done between circadian rhythm pathway index and LASSO gene index regarding the prognostic value of overall survival in glioma and LGG patients.

**Figure 6.**
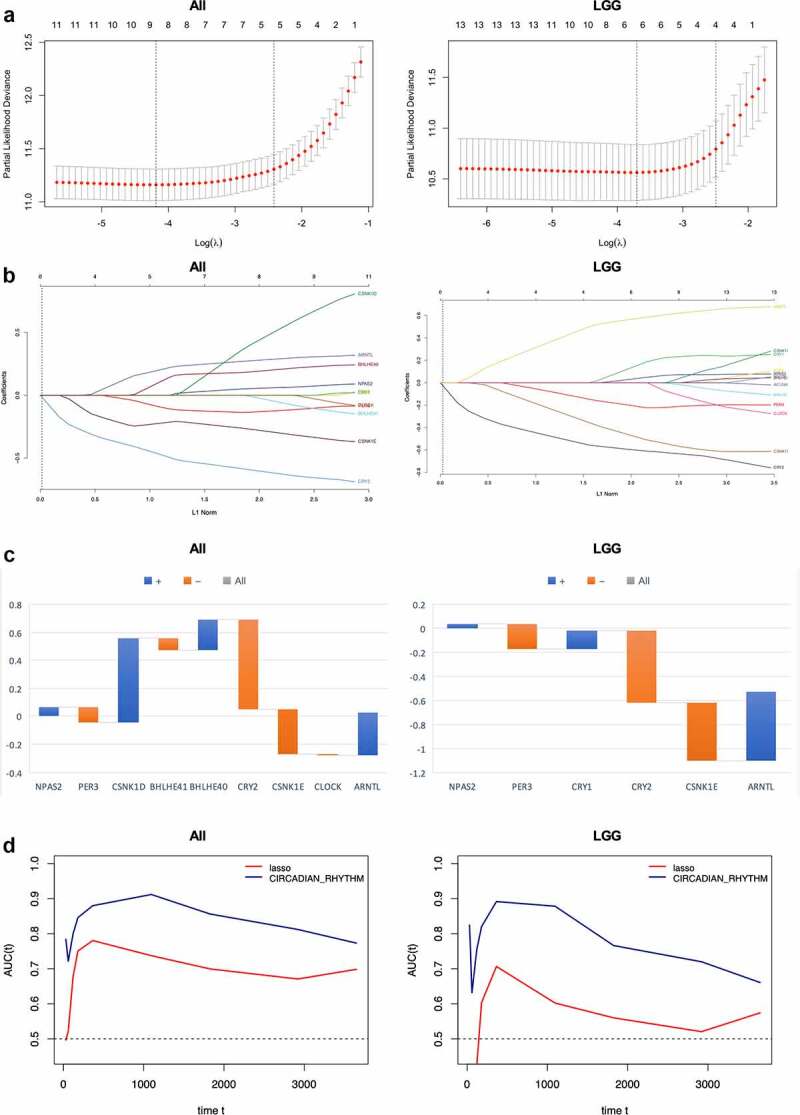
Prognostic value of circadian rhythm pathway index in glioma and LGG subgroup. (a). Cvfit plot for the selection of lambda value selection. The minimal lambda values were used for the construction of gene index. (b). LASSO regression of all 13 circadian rhythm pathway genes. (c). The gene index generated from LASSO regression. Orange: positive gene index; Blue: negative gene index. (d). TimeROC plot comparing the prognostic value of circadian rhythm pathway index and the LASSO regression index. Time points selected including 0, 30, 60, 120, 180, 365, 1095, 1825, 2920, 3650 days

Cvplots generated showed the minimal of lambda value in 9 and 6, respectively, in glioma and LGG patients (Figure 6(a)). LASSO regression results were shown in Figure 6(b). In all 13 genes enrolled, CRY2 showed the best survival significance in all patients as well as LGG subgroup, followed by CSNK1E and ARNTL, while in all patients has seen CSNK1E outdoing ARNTL, but reversed in LGG subgroup. Gene indexes generated were shown as below (Figure 6(c)):

All:

$$\begin{array}{rl} & GeneIndex=0.07*Expression\left(NPAS2\right)-0.11\\ & *Expression\left(PER3\right)+0.61*Expression\left(CSNK1D\right)\\ & -0.09*Expression\left(BHLHE41\right)+0.22\\ & *Expression\left(BHLHE40\right)-0.64\\ & *Expression\left(CRY2\right)-0.32*Expression\left(CSNK1E\right)\\ & -0.01*Expression\left(CLOCK\right)+0.30\\ & *Expression\left(ARNTL\right) \end{array}$$

LGG:

$$\begin{array}{rl} & GeneIndex=0.04*Expression\left(NPAS2\right)-0.21\\ & *Expression\left(PER3\right)+0.16*Expression\left(CRY1\right)\\ & -0.60*Expression\left(CRY2\right)-0.49*Expression\\ & \left(CSNK1E\right)+0.58*Expression\left(ARNTL\right) \end{array}$$

Comparisons were done using timeROC to evaluate the prognostic value of the two indexes with circadian pathway index. As shown in Figure 6(d), at each time point, the circadian pathway index exhibits higher ROC score compared to LASSO regression index, demonstrating better prognostic value of pathway index in glioma and LGG patients. In all glioma patients, the highest score was 0.91 seen at 3 years of follow-up, while in LGG patients, the highest score was 0.89, which was seen at 1 year. At the endpoint of 10 years, the ROC score remained 0.76 and 0.67 in all glioma patients and LGG patients, respectively.

## Discussion

Circadian rhythm pathway has demonstrated both clinical and molecular relevance with glioma, therefore, a comprehensive understanding of the underlying genetic influence and overall status of circadian rhythm pathway will enable us to understand the biological importance of circadian rhythm in glioma. By adopting bioinformatic methods, multi-omics landscape of circadian rhythm pathway was depicted and compared between LGG and GBM patients. The alteration of circadian rhythm pathway showed significantly higher level of alterations in LGG patients and the alteration of circadian rhythm pathway significantly correlated with clinical outcomes. Furthermore, the omics-alteration was positively correlated the expressional profile of circadian rhythm pathway genes. To quantify the alteration of circadian rhythm pathway, a pathway index was generated. In LGG patients, the circadian rhythm pathway was highly deregulated and significantly correlate with better survival outcomes, which was not seen in GBM patients. Our findings revealed the different biological functions of circadian rhythm pathway between LGG and GBM patients, and the construction of circadian rhythm pathway index was demonstrated good efficacy in quantifying the overall status of the pathway, which can serve as a putative prognostic biomarker for LGG patients.

Previously published researches focusing on each single genes on the circadian rhythm pathway presented varied conclusions, which barely reflecting the actual effects of alterations of circadian pathway in tumor patients [9]. By depicting a comprehensive multi-omics landscape of the pathway, we provide systematic insights into the biological function of circadian pathway in glioma, which was demonstrated different by pathological subtypes. LGG patients contain highly altered pathway than GBM patients and the clinical outcome of LGG patients was affected by the status of the pathway. Comparing to other studies, our results first revealed the difference between LGG and GBM patients. Previously published researched focusing on single-gene functions and also reported contradictory functions between cell lines or subtypes, however, failed to notice the difference from a more general perspective [37].

R package ‘Pathifier’ was developed to transform gene-level information into pathway-level information by inferring pathway deregulation scores for each tumor sample on the basis of expression data. By combining expression data from TCGA LGGGBM and GTEx brain dataset, alterations of circadian rhythm pathway were evaluated in comparison with the normal brain tissue, so as to reflect the actual status of the whole pathway. Moreover, explorations of clinical associations and putative biological functions can be further done based on the pathway index generated. According to results presented in our study, the pathway index was demonstrated a solid prognostic value in glioma, especially LGG patients. Referring to previous studies, overexpression of core clock genes, including PER2/3, CRY1/2, significantly correlated with better survival, while oncogenetic functions were seen in CLOCK, NPAS2 and BMAL1, which in accordance with our results [37,38]. From one perspective to another, bioinformatic analyses with large sample size and big data can unravel the correlation underlying, statistical significance obtained by big data has been demonstrated better reliability comparing to experiments done with less sample size. Numeration of abstract concepts enables scholars to explore the mechanism behind phenotypes more effectively [39–41].

Based on the clinical significance demonstrated in our study, the intervention of circadian rhythm pathway can work as a putative therapeutic target. Melatonin, the circadian hormone secreted by the pineal gland, was discussed therapeutic potency in numerous tumors [20]. In vivo analyses revealed pharmaceutic functions of melatonin as an anti-inflammatory and antioxidant agent [42], as well as an epigenetic regulator in glioma [43]. Melatonin in cancer initiation and progression also demonstrated various effects that can increase cellular detoxification regulate epigenetic modifications and further suppress tumor metabolism*^[44]^*, inhibit cancer proliferation, cell cycle and angiogenesis [43,45]. Melatonin used as an adjuvant intervention in the treatment of glioma was shown the ability to sensitize the chemotherapeutic response and attenuate the resistance process [46]. Clinical trials with melatonin as therapeutic intervention mainly seen in breast cancer. Melatonin use before and during the first cycle of adjuvant chemotherapy for breast cancer was demonstrated better effect at improving the descending pain modulatory system (DPMS) in a recent study published. These results suggest that oral melatonin, together with the first adjuvant chemotherapy for breast cancer counteracts the dysfunction in the inhibitory DPMS and improves pain perception measures. However, the anticancer effect of melatonin was rarely mentioned and need further research.

Considering the symptoms of glioma shown differs from tumor locations, we especially explored the relationship between tumor location and the activity of circadian rhythm pathway. The pathway index between tumor locations showed no difference on one-way ANOVA, while Tukey’s multiple comparisons test showing only marginal significant differential expression between frontal lobe and temporal lobe (0.449 ± 0.142 vs 0.406 ± 0.160, P = 0.0179). Given the anatomical basis of circadian rhythm, further analysis of supratentorial localization was done, with highest level of pathway index was in the cerebral cortex. However, subgroup survival analyses showed clinical association in all groups, higher score correlate with better survival outcomes in LGG patients. These results demonstrated that the circadian pathway index was irreverent with tumor location.

The poor prognosis of glioma requests urgent exploration of better prognostic biomarkers. Numerous works were done using multi-omics data. Current bioinformatic methods for estimating biomarkers showed discrepancies and new methods are been developed. LASSO regression is one of the methods that are most often used in dimension reduction and elements selection. Therefore, we also did LASSO regression in glioma and LGG patients to generate new indexes so as to compare with circadian pathway index generated in our study. Comparisons were made using timROC plot at several time point and pathway index has outdone LASSO index at every time point estimated, demonstrating good prognostic value. Further comparisons were made with previously reported biomarkers also using TCGA data. A five genes (DES, RANBP17, CLEC5A, HOXC11, POSTN) risk score was constructed using WGCNA and Cox regression with The AUC for 1, 3, 5-year-survival were 0.771, 0.808, and 0.838, respectively*^[47]^*; another work used combined elevation of AURKB and UBE2C to predict severe outcomes and therapy resistance in glioma, and the AUC reported were 0.875 and 0.849 [48]. In our study, the AUC for 1, 3, 5-year-survival were 0.85, 0.91, 0.85, respectively, and demonstrated better efficacy as a prognostic value in glioma.

Except for the aforementioned strengths and perspectives, this study has some limitations. As a bioinformatic analysis, results require further validation using experimental or clinical methods, which were not included in this study, therefore, the conclusion provided was limited to research hypotheses. Furthermore, the classification of glioma into LGG and GBM was coarse, considering the diagnosis of glioma required specific pathological subtypes and there might be difference between each subtype which were not discussed. Further explorations should be done to fully understand the correlation between circadian rhythm alteration and glioma.

## Conclusion

In conclusion, by adopting bioinformatic analysis, we generated a multi-omics landscape of circadian rhythm pathway in glioma and found that LGG patients containing highly altered circadian rhythm genes, which significantly correlated with the clinical outcome of LGG patients. Furthermore, the pathway index efficiently reflected the deregulative status of circadian rhythm pathway in glioma and was demonstrated of significant prognostic value in LGG patients.

## Supplementary Material

Supplemental Material

## Data Availability

The datasets generated and/or analysed during the current study are available in the UCSC XENA repository, [https://tcga.xenahubs.net]. Data used included the Cancer Genome Atlas (TCGA, http://can‑cergenome.nih.gov/), the GTEx projects.

## Highlights

1. Circadian rhythm pathway has higher frequencies of mutations in LGG than GBM;
2. Alterations in circadian rhythm pathway correlate with the prognosis of LGG patients;
3. Circadian rhythm pathway index can be used to quantify general alterations of the pathway;
4. Circadian rhythm pathway index significantly correlated with the prognosis of LGG patients.
