# Supplementary material for: Multi-omics landscape of circadian rhythm pathway alterations in Glioma
Source: Bioengineered. 2021 Jul 5;12(1):3294–308. doi: 10.1080/21655979.2021.1947075 (PMC8806853; doi:10.1080/21655979.2021.1947075)
Supplement: Supplemental Material [file KBIE_A_1947075_SM9219.zip › supplementary/downloadFromZipFile.pdf]

Figure S1. Figure legends of clinical annotations.

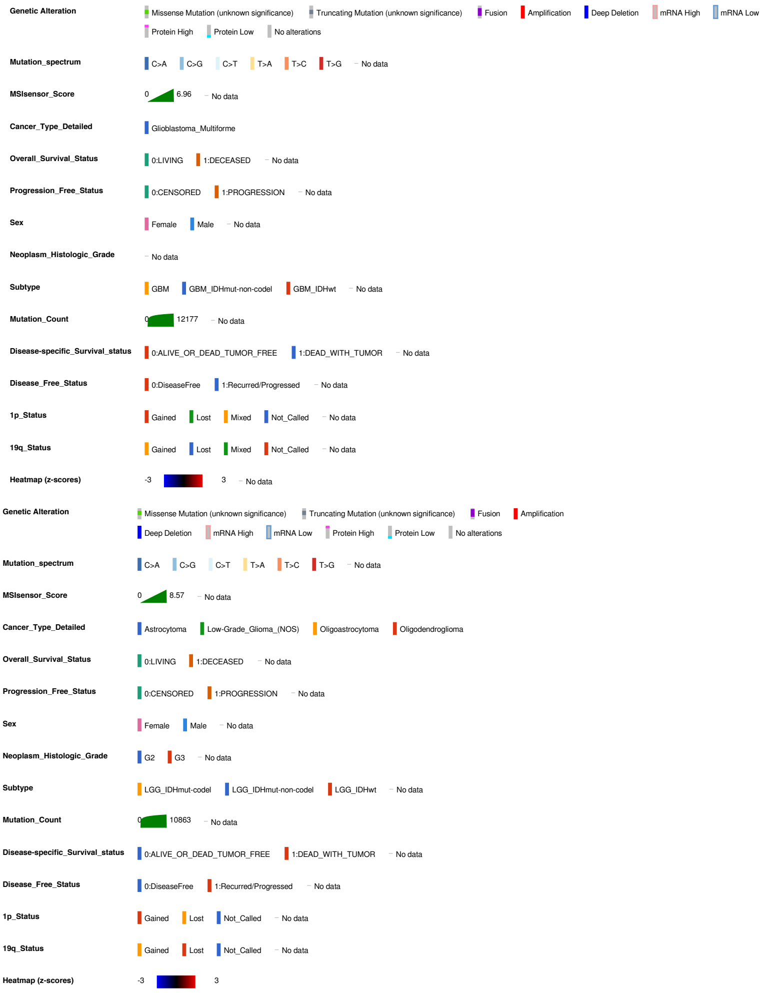

Table S1. Co-occurrence of gene mutations.

A	B	Neither	A Not B	B Not A	Both	Log2 Odds Ratio	p-Value	q-Value	Tendency
BTRC	PRKAA2	379	24	6	14	>3	<0.001	<0.001	Co-occurrence
BTRC	RORB	370	22	15	16	>3	<0.001	<0.001	Co-occurrence
BTRC	NPAS2	370	23	15	15	>3	<0.001	<0.001	Co-occurrence
CLOCK	RORB	377	15	19	12	>3	<0.001	<0.001	Co-occurrence
PRKAA2	RORB	382	10	21	10	>3	<0.001	<0.001	Co-occurrence
CUL1	RORB	324	68	13	18	2.722	<0.001	<0.001	Co-occurrence
NPAS2	NR1D1	387	23	6	7	>3	<0.001	<0.001	Co-occurrence
BTRC	PRKAA1	357	25	28	13	2.729	<0.001	<0.001	Co-occurrence
FBXL3	RBX1	370	39	6	8	>3	<0.001	0.001	Co-occurrence
NPAS2	PRKAA1	363	19	30	11	2.808	<0.001	0.001	Co-occurrence
BHLHE40	PER2	379	14	22	8	>3	<0.001	0.002	Co-occurrence
CUL1	NPAS2	322	71	15	15	2.181	<0.001	0.005	Co-occurrence
CUL1	PER2	322	71	15	15	2.181	<0.001	0.005	Co-occurrence
CRY1	CUL1	327	10	74	12	2.407	<0.001	0.008	Co-occurrence
CREB1	FBXL3	365	11	39	8	2.767	<0.001	0.011	Co-occurrence
BHLHE40	CRY1	385	16	16	6	>3	<0.001	0.011	Co-occurrence
BHLHE40	PRKAA1	368	14	33	8	2.672	<0.001	0.011	Co-occurrence
CUL1	PRKAA2	328	75	9	11	2.418	<0.001	0.011	Co-occurrence
CRY2	PRKAA1	370	12	34	7	2.666	0.001	0.023	Co-occurrence
BTRC	CUL1	315	22	70	16	1.71	0.001	0.024	Co-occurrence
PRKAA1	PRKAA2	369	34	13	7	2.547	0.001	0.028	Co-occurrence
NPAS2	PRKAA2	379	24	14	6	2.759	0.001	0.028	Co-occurrence
CUL1	PRKAG2	319	72	18	14	1.785	0.002	0.028	Co-occurrence
BHLHE40	BTRC	370	15	31	7	2.478	0.002	0.03	Co-occurrence
CREB1	CRY1	387	14	17	5	>3	0.002	0.03	Co-occurrence
FBXL3	FBXW11	356	38	20	9	2.076	0.002	0.035	Co-occurrence
PRKAG1	SKP1	376	28	13	6	2.632	0.002	0.035	Co-occurrence
BHLHE40	NPAS2	377	16	24	6	2.558	0.003	0.04	Co-occurrence

BHLHE40	NR1D1	392	18	9	4	>3	0.003	0.043	Co-occurrence
CSNK1E	NR1D1	378	32	8	5	2.884	0.003	0.043	Co-occurrence
ARNTL	RORB	376	16	25	6	2.496	0.003	0.043	Co-occurrence
BTRC	NR1D1	377	33	8	5	2.836	0.003	0.046	Co-occurrence
NPAS2	RORB	369	23	24	7	2.226	0.003	0.046	Co-occurrence

Table S2. Correlation between circadian rhythm pathway genes and pathway index.

Gene_1	Gene_2	All	Normal	LGG	GBM
CIRCADIAN_RHYTHM	NPAS2	-0.35	0.08	-0.48	-0.11
CIRCADIAN_RHYTHM	PER3	0.24	0.67	-0.03	0.32
CIRCADIAN_RHYTHM	PER2	0.33	0.77	-0.04	0.25
CIRCADIAN_RHYTHM	CSNK1D	0.41	0.77	0.05	-0.01
CIRCADIAN_RHYTHM	CRY1	0.24	0.58	0.01	-0.08
CIRCADIAN_RHYTHM	BHLHE41	-0.39	-0.22	-0.61	-0.41
CIRCADIAN_RHYTHM	BHLHE40	-0.52	-0.31	-0.64	-0.30
CIRCADIAN_RHYTHM	NR1D1	0.13	0.80	-0.19	-0.06
CIRCADIAN_RHYTHM	CRY2	0.40	0.81	0.27	0.24
CIRCADIAN_RHYTHM	CSNK1E	0.74	0.57	0.67	0.51
CIRCADIAN_RHYTHM	AC129492.1	0.13	0.24	0.13	0.18
CIRCADIAN_RHYTHM	CLOCK	0.19	0.68	-0.30	0.04
CIRCADIAN_RHYTHM	ARNTL	-0.29	0.63	-0.46	-0.60
NPAS2	PER3	0.01	-0.50	-0.20	0.06
NPAS2	PER2	0.14	0.03	0.17	0.30
NPAS2	CSNK1D	-0.24	-0.03	-0.03	0.34
NPAS2	CRY1	-0.44	-0.41	-0.12	-0.24
NPAS2	BHLHE41	-0.06	-0.55	0.11	0.34
NPAS2	BHLHE40	0.59	0.51	0.53	0.47
NPAS2	NR1D1	0.45	0.23	0.40	0.46
NPAS2	CRY2	0.35	0.38	0.05	0.12
NPAS2	CSNK1E	-0.44	-0.10	-0.43	-0.01
NPAS2	AC129492.1	0.05	0.05	0.02	0.10
NPAS2	CLOCK	0.05	-0.14	0.26	0.17
NPAS2	ARNTL	0.47	0.46	0.49	0.08
PER3	PER2	0.58	0.61	0.33	0.64
PER3	CSNK1D	0.16	0.53	0.05	0.24
PER3	CRY1	0.20	0.74	-0.06	-0.06

PER3	BHLHE41	0.32	0.33	0.40	0.24
PER3	BHLHE40	-0.11	-0.52	0.20	0.09
PER3	NR1D1	0.55	0.46	0.25	0.42
PER3	CRY2	0.58	0.35	0.37	0.51
PER3	CSNK1E	0.12	0.42	0.12	0.21
PER3	AC129492.1	0.10	0.13	0.04	0.14
PER3	CLOCK	0.51	0.60	0.33	0.28
PER3	ARNTL	0.00	0.13	-0.11	-0.19
PER2	CSNK1D	0.41	0.64	0.10	0.40
PER2	CRY1	0.32	0.58	0.10	-0.05
PER2	BHLHE41	0.04	-0.08	0.08	0.26
PER2	BHLHE40	0.02	-0.13	0.30	0.24
PER2	NR1D1	0.54	0.58	0.38	0.36
PER2	CRY2	0.62	0.63	0.51	0.56
PER2	CSNK1E	0.23	0.48	0.00	0.22
PER2	AC129492.1	0.16	0.18	0.13	0.15
PER2	CLOCK	0.47	0.54	0.22	0.27
PER2	ARNTL	0.16	0.41	0.03	-0.18
CSNK1D	CRY1	0.55	0.59	0.10	-0.03
CSNK1D	BHLHE41	-0.04	-0.05	-0.04	0.33
CSNK1D	BHLHE40	-0.23	-0.19	-0.01	0.41
CSNK1D	NR1D1	0.24	0.62	0.00	0.36
CSNK1D	CRY2	0.24	0.66	0.03	0.40
CSNK1D	CSNK1E	0.54	0.71	0.21	0.24
CSNK1D	AC129492.1	0.25	0.27	0.41	0.18
CSNK1D	CLOCK	0.30	0.51	-0.08	0.24
CSNK1D	ARNTL	0.17	0.50	0.03	0.02
CRY1	BHLHE41	0.08	0.33	-0.18	-0.07
CRY1	BHLHE40	-0.35	-0.43	-0.01	0.00
CRY1	NR1D1	0.02	0.39	-0.34	-0.24

CRY1	CRY2	-0.07	0.26	-0.17	-0.03
CRY1	CSNK1E	0.32	0.43	0.12	0.16
CRY1	AC129492.1	0.09	0.10	0.16	0.06
CRY1	CLOCK	0.30	0.54	0.17	-0.08
CRY1	ARNTL	0.14	0.25	0.17	0.25
BHLHE41	BHLHE40	0.24	-0.10	0.51	0.46
BHLHE41	NR1D1	0.04	-0.22	0.17	0.47
BHLHE41	CRY2	-0.13	-0.39	-0.14	0.22
BHLHE41	CSNK1E	-0.21	0.01	-0.36	-0.24
BHLHE41	AC129492.1	-0.04	0.04	-0.14	-0.04
BHLHE41	CLOCK	0.13	-0.02	0.27	0.22
BHLHE41	ARNTL	-0.01	-0.35	0.18	0.34
BHLHE40	NR1D1	0.21	-0.05	0.48	0.47
BHLHE40	CRY2	0.04	0.01	0.05	0.10
BHLHE40	CSNK1E	-0.42	-0.02	-0.51	-0.20
BHLHE40	AC129492.1	0.00	0.02	-0.07	-0.01
BHLHE40	CLOCK	-0.14	-0.48	0.35	0.23
BHLHE40	ARNTL	0.29	0.03	0.44	0.17
NR1D1	CRY2	0.70	0.73	0.56	0.35
NR1D1	CSNK1E	-0.03	0.44	-0.21	-0.08
NR1D1	AC129492.1	0.11	0.15	0.02	0.07
NR1D1	CLOCK	0.36	0.41	0.13	0.20
NR1D1	ARNTL	0.44	0.65	0.24	0.22
CRY2	CSNK1E	0.20	0.40	0.23	0.25
CRY2	AC129492.1	0.16	0.22	0.14	0.13
CRY2	CLOCK	0.37	0.41	0.10	0.19
CRY2	ARNTL	0.19	0.60	0.00	-0.03
CSNK1E	AC129492.1	0.18	0.26	0.27	0.24
CSNK1E	CLOCK	0.10	0.31	-0.19	-0.02
CSNK1E	ARNTL	-0.26	0.33	-0.33	-0.31

AC129492.1	CLOCK	0.09	0.16	-0.04	0.07
AC129492.1	ARNTL	0.09	0.19	-0.01	-0.18
CLOCK	ARNTL	0.30	0.50	0.39	-0.04
